# Ruptured intraventricular tuberculous brain abscess mimicking cystic neoplasm: a case report

**DOI:** 10.11604/pamj.2021.39.122.29369

**Published:** 2021-06-12

**Authors:** Zakariae Benyaich, Farouk Hajhoui, Mehdi Laghmari, Houssine Ghannane, Said Ait Benali

**Affiliations:** 1 Department of Neurosurgery, University Hospital Center Mohammed VI of Marrakech, Marrakech, Morocco,; 2 Faculty of Medicine and Pharmacy of Marrakech, Cadi Ayyad University, Marrakech, Morocco

**Keywords:** Tuberculosis, brain abscess, cerebral ventricles, intracranial hypertension, case report

## Abstract

Central nervous system (CNS) tuberculosis is a potentially life-threatening condition that may manifest in different forms and simulate other pathologies. It rarely involves the ventricles and the occurrence of primary intraventricular tuberculous brain abscess (TBA) has exceptionally been reported. As far as we know, ruptured intraventricular TBA has not been described before. An immunocompetent 56-years-old man was admitted for sub-acute intracranial hypertension with behaviour disorders. Cranial magnetic resonance imaging (MRI) showed a cystic lesion of the third ventricle containing fluid-fluid level with biventricular hydrocephalus and debris in the occipital horns. A ruptured cystic neoplasm was first considered. The patient underwent surgery via a right transcortical transventricular approach, combining both microscope and endoscope. The puncture of the lesion brought pus and the Ziehl-Neelson (ZN) staining demonstrated acid-fast bacilli. Intraventricular tuberculous abscess is an extremely rare condition that can take an unusual radiological appearance. This observation highlights the consideration of tuberculosis within the list of differential diagnosis of intraventricular cystic lesions in immunocompetent hosts.

## Introduction

Central nervous system (CNS) tuberculosis is one of the most devastating manifestations of tuberculosis and a leading cause of morbidity in developing countries. Its main pathological manifestations include meningoencephalitis, tuberculomas, and less frequently, abscesses [1]. The involvement of ventricles is rare and the occurrence of intraventricular tuberculous abscess has been reported in literature only one time so far [2]. Thus, without any specific clinical and radiological signs, the diagnosis of such condition can be very challenging. In this report, we describe the case of a ruptured tubercular abscess of the third ventricle in a 56-year-old man presenting with an atypical radiological image mistaken for a ventricular cystic tumor.

## Patient and observation

**Patient information:** a 56-years-old man, without any pathological background, presented with a one-month history of walk imbalance, urinary incontinence, and signs of increased intracranial pressure consisting of intense headaches, vomiting, and episodes of brief consciousness loss.

**Clinical findings:** the physical examination found a conscious patient, afebrile, with gait apraxia and distal postural tremor. The cranial nerves examination was normal, and neither motor nor sensory deficits were detected.

**Figure 1:**
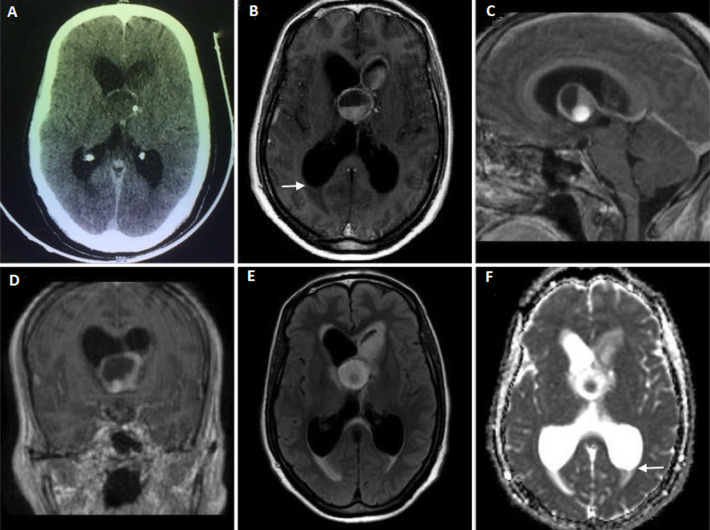
contrasted head CT scan; A) showing an isodense mass within the third ventricle with mild rim contrast enhancement and spot calcification. Contrasted axial; B) sagittal C) coronal; D) T1-weighted MR images showing a bilobate cystic lesion encompassing the third ventricle and the left frontal horn with fluid-fluid level and mild rim contrast enhancement; this lesion contains both hypointense and hyperintense components in FLAIR; E) and in apparent diffusion coefficient map; F) note the debris in the occipital horns of the lateral ventricles with a fluid-fluid level (white arrow)

**Diagnostic assessment:** a cranial computed tomography scan (CT scan) revealed an isodense mass within the third ventricle obstructing the foramen of Monro bilaterally and extending to the left frontal horn with a mild rim contrast enhancement and small calcifications, associated with an obstructive hydrocephalus (Figure 1). Magnetic resonance imaging (MRI) disclosed a bilobate lesion including a cystic part of 23mm of diameter extending from the third ventricle to both of the foramen of Monro and a second heterogenous part in the left frontal horn of the lateral ventricle. The cystic part in the third ventricle contained three components on high, middle and low signal intensity on both T1 and T2-weighted images with a fluid-fluid level. There was a mild peripheral enhancement after gadolinium administration. In addition to the obstructive hydrocephalus, there were deposits in the occipital horns of the lateral ventricles (Figure 1). Apart from a deep hyponatremia of 117mEq/l and a quantitative C-reactive protein of 15mg/L, baseline blood investigations were within normal range. The chest X-ray was unremarkable. Based on clinical and radiological grounds, a presumptive diagnosis of cystic neoplasm, as colloid cyst or craniopharyngioma, was initially considered.

**Figure 2:**
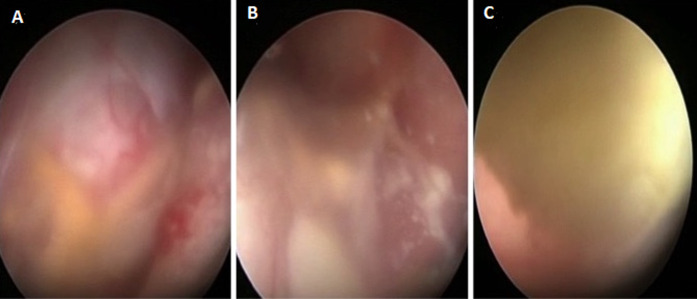
A) intra-operative endoscopic view of the lesion encompassing the foramen of Monro; B) signs of ventriculitis; C) and a cluster of pus in the frontal horn of the left lateral ventricle

**Figure 3:**
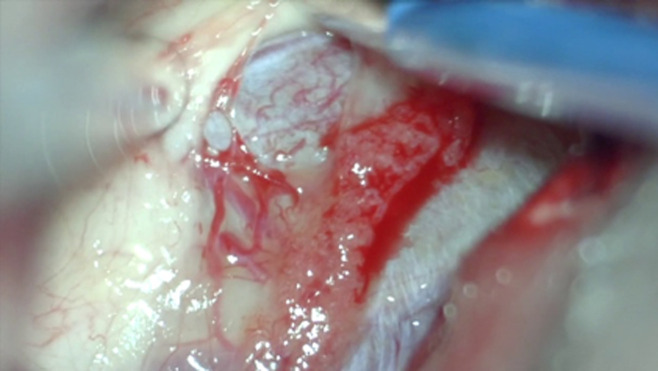
intra-operative image of the lesion seen through a right transcortical transventricular approach

**Therapeutic intervention:** right frontal craniotomy, transcortical transventricular approach to the lesion was performed one week after admission, combining both microscope and endoscope. On endoscopic visualization, the lesion was spherical, extending from the septum pellucidum to the third ventricle and encompassing the foramens of Monro (Figure 2). There was a yellowish and gelatinous fluid inside the ventricles. After microsurgical cauterization and opening of the cystic wall that was highly vascularized, a yellowish pus was evacuated by puncture and aspiration. The lesion was highly adherent to the fornix, preventing a complete excision of the wall (Figure 3). The part of the lesion within the left frontal horn was also punctured through the septum pellucidum with the help of the endoscope. After washing out the ventricles, an external ventricular drain was left on the right lateral ventricle. On bacterial examination of the pus, ZN staining demonstrated acid-fast bacilli (AFB). The histopathological examination of the cystic wall revealed vascular granulations with non-specific inflammatory reaction. GeneXpert was positive for mycobacterium tuberculosis. HIV serology, performed after this finding, was negative. The antituberculosis therapy was started immediately, including rifampicin, isoniazid, pyrazinamide and ethambutol, as recommended by the national guidelines.

**Figure 4:**
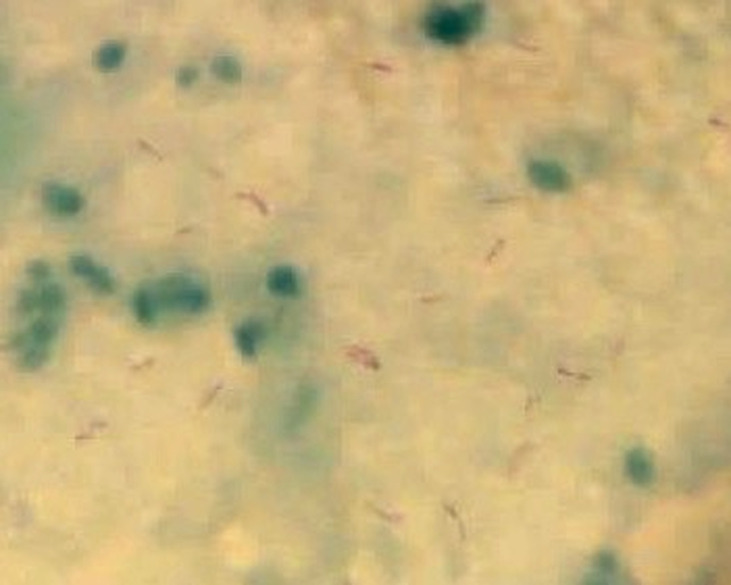
microscopic examination of the pus with ZN staining demonstrating many acid-fast bacilli

**Follow-up and outcomes:** post-operatively, the patient experienced a rapid neurologic improvement. The external ventricular drain was removed after seven days, and the patient was discharged from hospital ten days after surgery. Considering the risk of ventricular septation inherent to central nervous system (CNS) tuberculosis and recurrence of hydrocephalus, the patient and his family were advised to consult at the slightest worsening, in order to perform a ventriculoperitoneal shunt if necessary. But the patient was almost symptom-free at the first month follow-up examination, with only a mild memory disturbance, and the control blood tests were normal. Three months later, the patient was hospitalized again in another institution for a jaundice and altered general state. A drug-induced fulminant hepatitis was suspected, and unfortunately, he died a few days later.

## Discussion

Tuberculous brain abscess (TBA) is an uncommon lesion of the brain with an incidence of only 4-8% among immunocompetent patients with CNS tuberculosis [1], but in 20% of HIV-infected patients with CNS tuberculosis [3]. It is defined as an encapsulated collection of frank pus containing viable tubercular bacilli, without evidence of tubercular granuloma. Unlike tuberculomas, epithelioid cell reaction and giant cells are lacking in the abscess wall [4]. Tuberculous brain abscess develops usually intraparenchymal supratentorial [5], and primary intra-ventricular location has been reported in the literature only once so far, by Vajramani in 1999 [2]. According to our knowledge, no similar case of ruptured intraventricular TBA has been described before. The origin of such lesions is not clear. The tubercle bacilli could possibly reach the ventricles via hematogenous spread from a primary infection site, most often the lungs, through the choroid plexus [2]. In our case, the choroid plexus appeared inflamed and involved by the abscess on intra-operative view (Figure 3), suggesting that a previous choroid plexitis might have been the origin of the abscess formation. It is postulated that the type of the pathological lesion depends on various factors, including the immune host system, the size of the inoculum, and whether chemotherapy was given [6]. The development of a tuberculous abscess can be due to a large inoculum that lead to an excessive exudative reaction with massive caseation. The softening of the caseum with the influx of polymorphonuclear leucocytes leads to pus formation [7]. In immunocompromised individuals, a poor inflammatory response secondary to altered cell-mediated immunity may also result in abscess formation [3]. The usual radiological aspect of TBA closely resembles to pyogenic brain abscess. Both appear on CT scan images as a hypodense round or multiloculated lesion surrounded by a rim of contrast enhancement and a perifocal oedema [8]. Likewise, on conventional MRI, the center is hypointense on T1-weighted images, hyperintense on T2-weighted images with restriction on diffusion, and shows a rim enhancement on post-contrast T1-weighted images.

However, the significantly lower magnetization transfer value and the absence of amino acid peak at 0.9ppm on magnetic resonance MR spectroscopy can help to distinguish TBA from pyogenic abscess [9]. Tuberculous brain abscess can also be differentiated from other ring enhancing lesions by its typical prominent lipid peak on proton MR spectroscopy [10]. In our case, the abscess had an unusual cystic aspect, with fluid-fluid level and triple component hypo, middle and high signal intensity on T1 and T2 weighted MRI, and the restriction on diffusion was limited to the inferior part of the lesion, which made the diagnosis more challenging (Figure 1). This atypical presentation led us to consider first an intraventricular cystic neoplasm, like colloid cyst of the third ventricle. This later, in rare cases, may rupture into the ventricle and take a similar aspect with a mixed signal intensity inside the cyst and a fluid level in occipital horns [11]. But in retrospect, there was an enhancement of the ependymal wall of the left frontal horn evoking an ependymitis, which might have suggested an infectious process. The most probable cause of these atypical features is that the abscess ruptured into the ventricles and pus mixed with cerebrospinal fluid inside the cavity. It is postulated that a close proximity of the abscess (intraventricular in our case), along with adjacent ependymal enhancement, and the presence of debris in the dependent lateral ventricles (in both occipital horns in our case), are tell-tale signs of intraventricular rupture of brain abscess [12]. Mostly, direct communication between the abscess cavity and the ventricles is not obvious. Our patient met the diagnosis criteria for TBA according to Whitener, which are: macroscopic evidence of true abscess formation within the brain; demonstration of AFB in the pus or the abscess wall or mycobacterium tuberculosis on pus culture; and presence of inflammatory reaction composed predominantly of vascular granulation tissue containing acute and chronic inflammatory cells on the histological study [4]. If the diagnosis of TBA had been suspected before surgery, a less invasive procedure could have been discussed, such as a purely endoscopic approach or stereotactic puncture associated with an external ventricular drainage.

## Conclusion

Through this observation, despite its rarity, tuberculous brain abscess should be considered as a differential diagnosis of cystic lesions of the third ventricle, especially in endemic areas. We recommend, by extension, especially in those regions or in immunocompromised patients, an assessment of the tuberculous status of any patient with atypical brain lesion.
